# Two new species of the *Clubiona corticalis*-group from Yunnan Province, China (Araneae, Clubionidae)

**DOI:** 10.3897/zookeys.496.9223

**Published:** 2015-04-16

**Authors:** Pan-Long Wu, Guo Zheng, Feng Zhang

**Affiliations:** 1 The Key Laboratory of Invertebrate Systematics and Application, College of Life Sciences, Hebei University, Baoding, Hebei 071002, P. R. China; 2 College of Life Science and Chemistry, Shenyang Normal University, Shenyang, Liaoning 110034, P. R. China

**Keywords:** Sac spiders, *Paraclubiona*, *Atalia*, taxonomy, South-East Asia

## Abstract

The present paper describes two new *Clubiona corticalis*-group species collected from Xishuangbanna, Yunnan Province of China: *Clubiona submoralis* **sp. n.** (♀♂) and *Clubiona pollicaris* **sp. n.** (♀♂).

## Introduction

*Clubiona* Latreille, 1804, the largest genus of the Clubionidae, currently includes 468 species (Platnick 2014) widely distributed around the world (except South America). Because of its high species diversity, several revisions have been published by Simon (1932) for the French species, Lohmander (1944) for Swedish species, Wiehle (1965) for German species, Wunderlich (2011) for European species, Edwards (1958) for the North American species, Dondale and Redner (1982) for Canadian and Alaskan species, Mikhailov (1990, 1991, 1995, 2002, 2012) for Palaearctic species, and Deeleman-Reinhold (2001) for southeast Asian species.

*Clubiona corticalis*-group was first recognized by Simon (1932). *Atalia* Thorell, 1887 (type species *Atalia concinna* Thorell, 1887, belongs to the *corticalis*-group) and subgenus *Paraclubiona* Lohmander, 1944 (with type species *Clubiona corticalis*) are currently considered as junior synonyms of *Clubiona*. Species of this group can be recognized by the following characters: inflated tegulum; long filiform, spiniform, or short embolus; simple and weakly developed retrolateral tibial apophysis; the anterior position of copulatory openings (Mikhailov 1995).

Currently, the *corticalis*-group includes 48 species mainly distributed in Eurasia and Australia (Mikhailov 1995, Deeleman-Reinhold 2001, Liu et al. 2007). Among these, at least 18 species have been recorded from China (see Table 1).

**Table 1.** A list of *Clubiona corticalis*-group species in China.

	Species name	Known sex	Distribution
1	*Clubiona altissimoides* Liu et al., 2007	♂♀	Yunnan
2	*Clubiona applanata* Liu et al., 2007	♂♀	Yunnan
3	*Clubiona brachyptera* Zhu, Ren & Chen, 2012	♂♀	Hainan
4	*Clubiona cordata* Zhang & Zhu, 2009	♂♀	Sichuan, Xizang
5	*Clubiona cylindrata* Liu et al., 2007	♂♀	Yunnan
6	*Clubiona didentata* Zhang & Yin, 1998	♂	Yunnan
7	*Clubiona kurosawai* Ono, 1986	♂♀	Taiwan
8	*Clubiona lamina* Zhang, Zhu & Song, 2007	♂	Yunnan
9	*Clubiona lyriformis* Song & Zhu, 1991	♀	Hubei
10	*Clubiona moralis* Song & Zhu, 1991	♂♀	Hubei
11	*Clubiona parallela* Hu & Li, 1987	♂♀	Xizang
12	*Clubiona pyrifera* Schenkel, 1936	♂♀	Gansu
13	*Clubiona qiyunensis* Xu, Yang & Song, 2003	♂♀	Fujian, Anhui
14	*Clubiona taiwanica* Ono, 1994	♂♀	Yunnan, Taiwan
15	*Clubiona tengchong* Zhang, Zhu & Song, 2007	♂	Yunnan
16	*Clubiona yaginumai* Hayashi, 1989	♂♀	Taiwan
17	*Clubiona submoralis* sp. n.	♂♀	Yunnan
18	*Clubiona pollicaris* sp. n.	♂♀	Yunnan

While examining *Clubiona* specimens collected from Xishuangbanna Prefecture, Yunnan Province of China, we found two new species belonging to the *corticalis*-group, which are described in this paper.

## Material and methods

All specimens studied are stored in 75% ethanol and deposited in the Institute of Zoology, Chinese Academy of Sciences in Beijing (IZCAS). All specimens were examined under a Tech XTL-II stereomicroscope. The photos, drawings and measurements were prepared using a Leica M205A stereomicroscope equipped with a DFC450 CCD camera and a drawing tube. Carapace length was measured from the anterior margin to the posterior margin of the carapace medially. The eyes were measured as the maximum diameter of the lens in dorsal or frontal view. The measurements of legs are shown as total length (femur, patella, tibia, metatarsus, tarsus). The epigyne was cleared in a solution of potassium hydroxide (KOH) and transferred to 75% ethanol for taking photos, drawing and measuring. All measurements are in millimeters.

The following abbreviations are used: ALE, anterior lateral eyes; AME, anterior median eyes; B, bursae; C, conductor; CO, copulatory openings; E, embolus; FD, fertilization ducts; MOA, median ocular area; PLE, posterior lateral eyes; PME, posterior median eyes; PPA, prolateral patellar apophysis; RFA, retrolateral femoral apophysis; RPA, retrolateral patellar apophysis; RTA, retrolateral tibial apophysis; S, spermathecae.

## Taxonomy

### Clubiona submoralis sp. n.

Taxon classificationAnimaliaAraneaeClubionidae

http://zoobank.org/3299A436-1170-4959-9BE3-95190B84F461

Figs 1–7 , 8–12

#### Type material.

Holotype ♂, CHINA, Yunnan Province, Xishuangbanna Prefecture, Mengla County, Menglun Town, XTBG (Xishuangbanna Tropical Botanical Garden), Yunnan Rubber Plantation (575 m; 21°54.46'N, 101°15.98'E), 21 July 2007, Guo Zheng leg. Paratypes: 9 ♂, 10 ♀, same data as holotype.

#### Diagnosis.

Among the Chinese species of the *corticalis*-group, this new species resembles *Clubiona moralis*, but differs by: a wider and triangular embolus (filiform and coiled in *Clubiona moralis*), presence of only one round, black marking on tegulum (several fan-shaped markings in *Clubiona moralis*); fertilization ducts opening in the medio-ventral side of the spermathecae (anteriorly in *Clubiona moralis*); and the spermathecae almost as large as the bursae (the bursae twice longer than the spermathecae in *Clubiona moralis*) (Figs 3–12).

**Figures 1–7.**
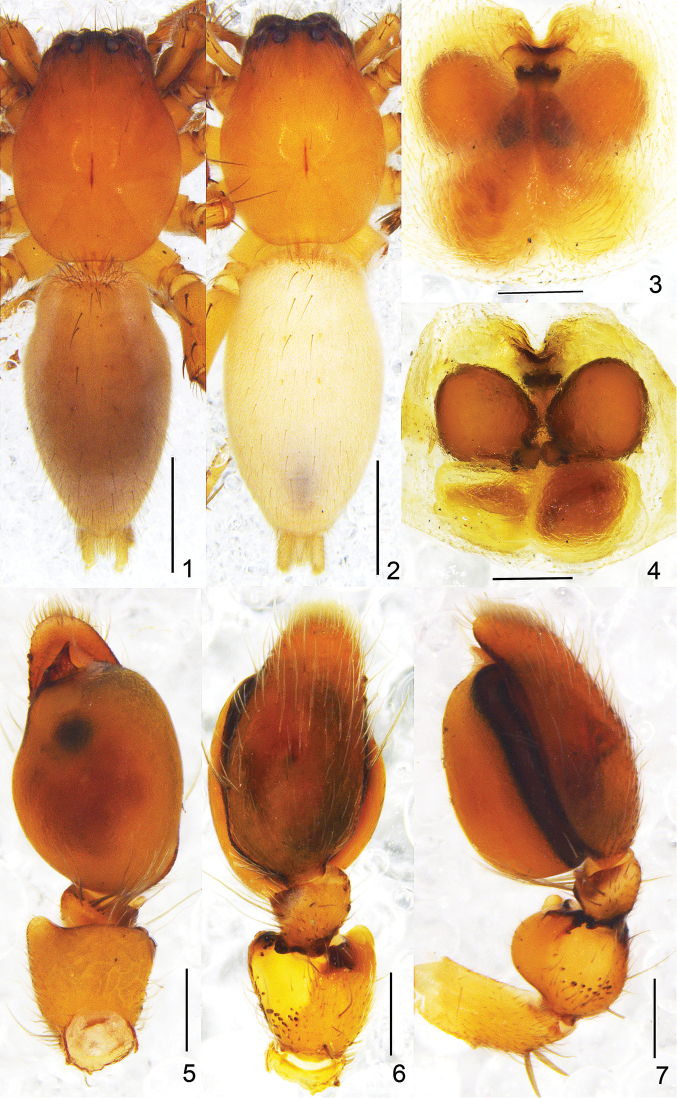
*Clubiona submoralis* sp. n. **1** male habitus, dorsal view **2** female habitus, dorsal view **3** epigyne, ventral view **4** vulva **5** left male palp, ventral view **6** same, dorsal view, showing tibial apophysis **7** same, retrolateral view. Scale bars: 1 mm (**1–2**); 0.2 mm (**3–7**).

**Figures 8–12.**
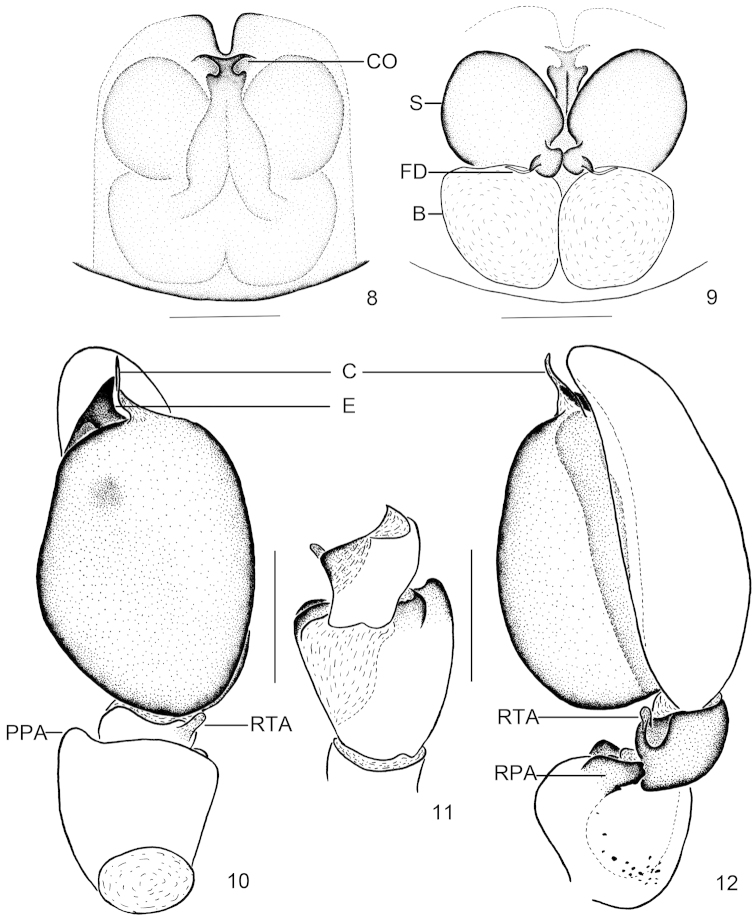
*Clubiona submoralis* sp. n. **8** epigyne, ventral view **9** vulva **10** left male palp, ventral view **11** tibial apophysis, dorsal view **12** left male palp, retrolateral view. Scale bars: 0.25 mm (**8–12**).

#### Etymology.

The species name refers to a close resemblance between the new species and *Clubiona moralis*.

#### Description.

Male. Total length 4.36–4.86. Holotype (Fig. 1): body 4.73 long; carapace 2.14 long, 1.96 wide; abdomen 2.49 long, 1.30 wide. Carapace brown. Median furrow longitudinal. In dorsal view, anterior eye row recurved, posterior eye row recurved. Eye sizes and interdistances: AME 0.10, ALE 0.11, PME 0.11, PLE 0.10; AME–AME 0.07, AME–ALE 0.06, PME–PME 0.27, PME–PLE 0.15. MOA 0.31 long, front 0.30 wide, back 0.48 wide. Chelicerae dark brown, promargin with six teeth, retromargin with three teeth. Endites brown, longer than wide. Labium dark brown, longer than wide. Sternum 1.19 long, 0.80 wide. Abdomen oval, brown, with conspicuous anterior tufts of hairs, dorsum with fine brown hairs. Legs yellow, both tibia I and II with two pairs of ventral spines, both metatarsi I and II with one pair of ventral spines. Measurements of legs: I 5.50 (1.55, 0.75, 1.57, 1.07, 0.56), II 6.02 (1.65, 0.80, 1.80, 1.21, 0.56), III 4.73 (1.26, 0.61, 1.13, 1.30, 0.43), IV 6.73 (1.78, 0.71, 1.65, 2.01, 0.58).

Palp (Figs 5–7, 10–12). Femur unmodified; patella swollen, almost globular, twice wider than tibia, and 1.5 wider than femur, with short and rounded pro- and retrolateral apophyses, retrolateral side with short modified spines near the base; tibia with small retro-ventral membranous apophysis. Cymbium longer than tegulum. Tegulum inflated, with a round, black marking medially; embolus short, wide, almost triangular in ventral view; conductor membranous, folded in the middle position, and almost threefold longer than wide.

Female. Total length 4.45–4.92. One paratype (Fig. 2) measured, body 4.47 long; carapace 1.87 long, 1.43 wide; abdomen 2.49 long, 1.39 wide. Eye sizes and interdistances: AME 0.10, ALE 0.11, PME 0.09, PLE 0.07; AME–AME 0.08, AME–ALE 0.04, PME–PME 0.24, PME–PLE 0.14. MOA 0.22 long, front 0.23 wide, back 0.41 wide. Sternum 0.96 long, 0.70 wide. Measurements of legs: I 4.53 (1.31, 0.66, 1.21, 0.84, 0.51), II 5.14 (1.48, 0.74, 1.41, 0.97, 0.54), III 4.22 (1.25, 0.57, 0.90, 1.13, 0.37), IV 6.14 (1.66, 0.70, 1.40, 1.89, 0.49). Coloration lighter than in male. Other characters as in male.

Epigyne (Figs 3–4, 8–9). Copulatory openings located anteriorly; in ventral view, the anterior part of copulatory ducts well visible and extending posteriorly, then connecting to bursae; spermathecae located anterior to bursae, both of them almost spherical, and with the same size as bursae.

#### Distribution.

China (Yunnan).

### Clubiona pollicaris sp. n.

Taxon classificationAnimaliaAraneaeClubionidae

http://zoobank.org/A815DA83-1925-4B07-BB84-D2DC0D5DA3C8

Figs 13–16 , 17–19 , 23–27

#### Type material.

Holotype ♂, CHINA, Yunnan Province, Xishuangbanna Prefecture, Mengla County, Menglun Nature Reserve (710 m; 21°57.70'N, 101°11.89'E), 7 August 2007, Guo Zheng leg. Paratypes: 6 ♂, 6 ♀, same data as holotype.

#### Diagnosis.

The new species differs from all other *Clubiona* species by a very long retrolateral femoral apophysis (almost as long as femur) and resembles *Clubiona qiyunensis* (Figs 20–22; also see Wu and Zhang (2014): 211, f. 13–23), but differs by: a smaller and inconspicuous embolus; the much longer femoral apophysis (in *Clubiona qiyunensis*, femoral apophysis is short, less than diameter of femur); a thumb-shaped prolateral patellar apophysis; anteriorly situated spermathecae; and rectangular bursae (Figs 15–19, 23–27).

**Figures 13–16.**
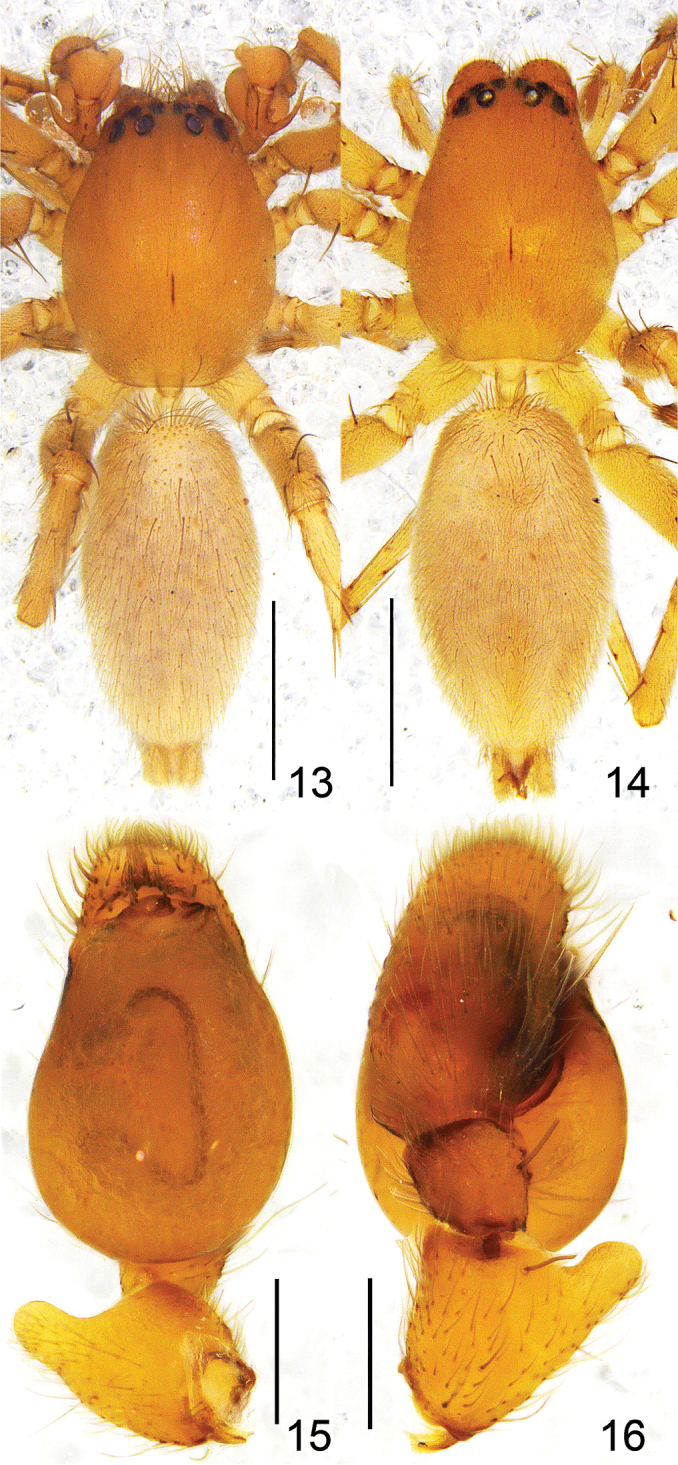
*Clubiona pollicaris* sp. n. **13** male habitus, dorsal view **14** female habitus, dorsal view **15** left male palp, ventral view **16** same, dorsal view, showing patellar apophysis. Scale bars: 1 mm (**13–14**); 0.2 mm (**15, 16**).

**Figures 17–22.**
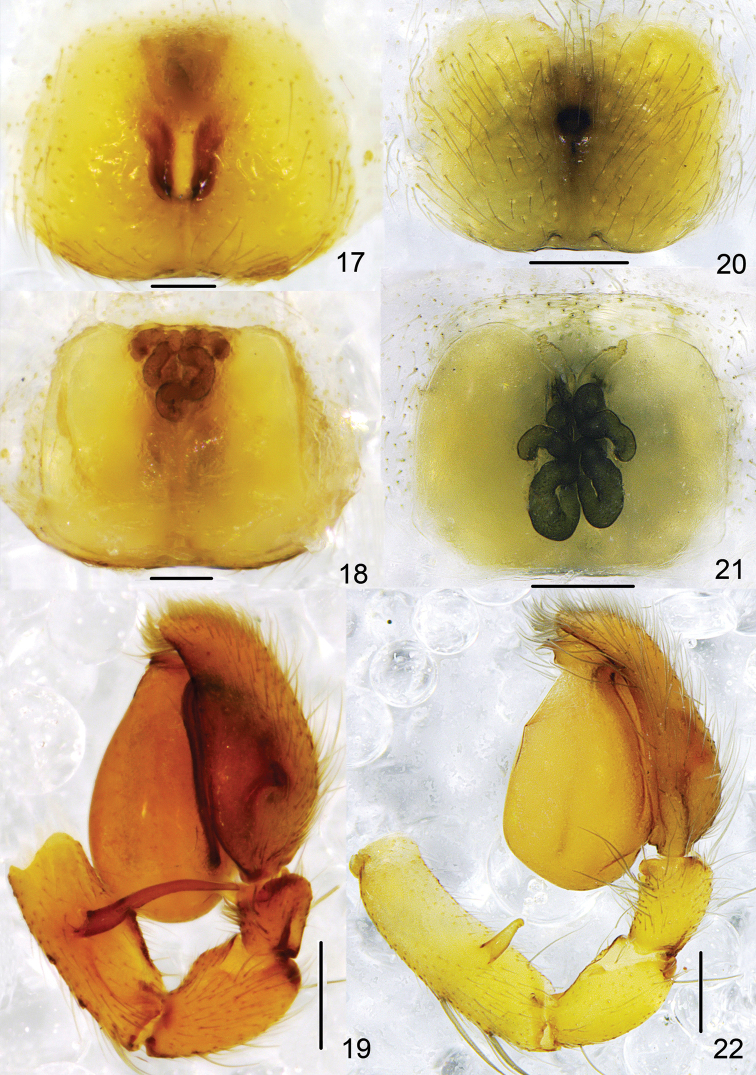
**17–19** *Clubiona pollicaris* sp. n. **17** epigyne, ventral view **18** vulva **19** left male palp, retrolateral view; **20–22** *Clubiona qiyunensis* Xu, Yang & Song, 2003. **20** epigyne, ventral view **21** vulva **22** left male palp, retrolateral view. Scale bars: 0.1 mm (**17, 18**) 0.2 mm (**19–22**).

**Figures 23–27.**
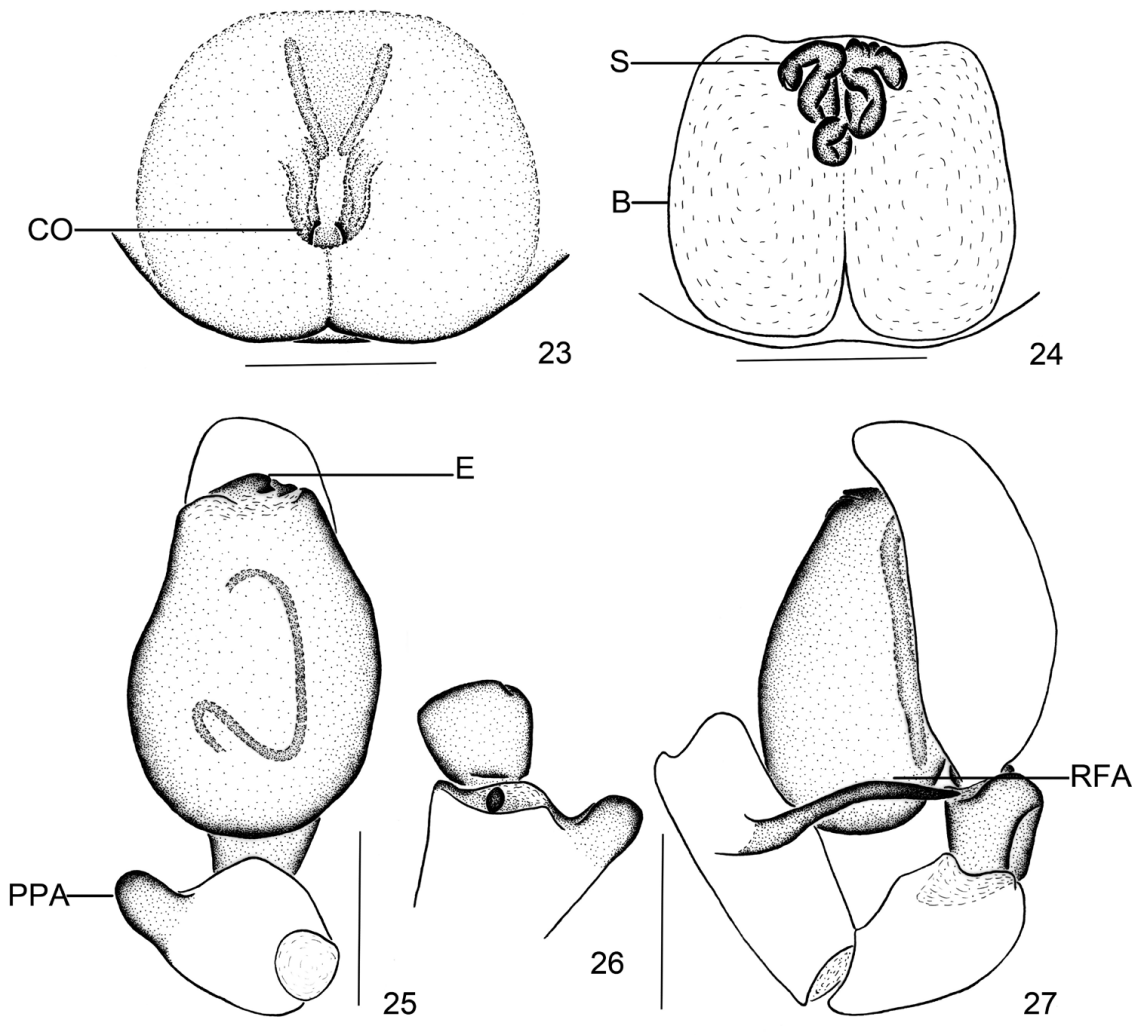
*Clubiona pollicaris* sp. n. **23** epigyne, ventral view **24** vulva **25** left male palp, ventral view **26** patellar apophysis, dorsal view **27** left male palp, retrolateral view. Scale bars: 0.25 mm (**23–27**).

#### Etymology.

The species name is derived from the Latin word “*pollicaris*”, and refers to the prolateral patellar apophysis, which in ventral view is thumb-shaped.

#### Description.

Male. Total length 3.20–3.49. Holotype (Fig. 13) body 3.27 long; carapace 1.51 long, 1.45 wide; abdomen 1.61 long, 0.90 wide. Carapace brown. Median furrow longitudinal. Both anterior and posterior eye row recurved in dorsal view. Eye sizes and interdistances: AME 0.06, ALE 0.10, PME 0.09, PLE 0.08; AME–AME 0.06, AME–ALE 0.03, PME–PME 0.19, PME–PLE 0.08. MOA 0.21 long, front 0.19 wide, back 0.37 wide. Chelicerae brown, promargin with five teeth, retromargin with four teeth. Both endites and labium brown, longer than wide. Sternum 0.83 long, 0.56 wide. Abdomen oval, brown, with conspicuous anterior tufts of hairs, dorsum with fine yellow hairs. Legs brown, both tibia I and II with two pairs of ventral spines, both metatarsi I and II with one pair of ventral spines. Measurements of legs: I 3.09 (0.84, 0.47, 0.83, 0.62, 0.33), II 3.46 (0.90, 0.50, 1.02, 0.69, 0.35), III 2.92 (0.84, 0.43, 0.63, 0.76, 0.26), IV 4.11 (1.18, 0.50, 0.96, 1.15, 0.32).

Palp (Figs 15–16, 19, 25–27). Femur modified, with long and thin retrolateral apophysis originating from median part, apophysis longer than tibia and subequal in length to femur; patella twice longer and 1.5 wider than tibia, with a round apophysis and thumb-shaped prolateral apophysis; tibia without apophyses; cymbium shorter than tegulum; tegulum inflated; sperm duct obvious, almost straight in retrolateral view; embolus short, originating from the apical tegulum prolaterally, directed almost horizontally.

Female. Total length 3.12–3.92. One paratype (Fig. 14) measured: body 3.90 long, carapace 1.52 long, 1.13 wide; abdomen 2.09 long, 1.18 wide. Eye sizes and interdistances: AME 0.07, ALE 0.09, PME 0.08, PLE 0.07; AME–AME 0.08, AME–ALE 0.05, PME–PME 0.20, PME–PLE 0.12. MOA 0.21 long, front 0.20 wide, back 0.35 wide. Sternum 0.85 long, 0.57 wide. Measurements of legs: I 2.69 (0.84, 0.38, 0.64, 0.51, 0.32), II 3.09 (0.90, 0.46, 0.79, 0.60, 0.34), III 2.63 (0.80, 0.35, 0.55, 0.67, 0.26), IV 3.92 (1.02, 0.50, 0.93, 1.10, 0.37). Coloration slightly lighter than in male; other characters as in male.

Epigyne (Figs 17–18, 23–24). Copulatory openings small, located almost in the centre of the epigynal plate; in ventral view copulatory ducts inconspicuous; spermathecae long, tubular and sinuous; bursae large, membranous, almost rectangular.

#### Distribution.

China (Yunnan).

#### Comments.

Retrolateral femoral apophysis is known only in three species of Clubionidae: *Clubiona pollicaris*, *Clubiona qiyunensis* and *Clubiona brachyptera*.

## Supplementary Material

XML Treatment for Clubiona submoralis

XML Treatment for Clubiona pollicaris
